# Optimization of fermentation conditions to increase the production of antifungal metabolites from *Streptomyces* sp. KN37

**DOI:** 10.1186/s12934-025-02652-w

**Published:** 2025-01-20

**Authors:** Xiaoyue Yang, Lijing Yuan, Muhammad Zeeshan, Chuntian Yang, Wen Gao, Guoqiang Zhang, Chunjuan Wang

**Affiliations:** 1 The Key Laboratory of Oasis Agricultural Pest Management and Plant Protection Utilization, College of Agriculture, Shihezi University, Shihezi, Xinjiang 832003 China; 2 College of Agriculture/Key Laboratory of Oasis Agricultural Pest Management and Plant Protection Resources Utilization, Shihezi University, Shihezi, Xinjiang 832003 China

**Keywords:** *Streptomyces* sp. KN37, Fermentation condition optimization, Plackett-burman design, Central composite design

## Abstract

The bacterium *Streptomyces* sp. KN37 was isolated from the soil of Kanas, Xinjiang. The broth dilution of strain KN37 has a strong inhibitory effect against a variety of crop pathogenic fungi. However, in practical applications, its effective biological activity is limited by medium formulations and fermentation conditions. In this study, we used the response surface method to optimize the fermentation medium and conditions of the strain KN37, for investigating the reasons for the enhanced biological activity at both the metabolic and transcriptomic levels. The results of the Plackett-Burman design showed that millet, yeast extract, and K_2_HPO_4_ were the key factors influencing its antifungal activity. Subsequently, optimization by the response surface methodology yielded the final fermentation conditions as: millet 20 g/L, yeast extract 1 g/L, K_2_HPO_4_ 0.5 g/L, rotation speed 150 r/min, temperature 25 °C, initial pH 8, fermentation time 9 d, inoculation amount 4%, liquid volume 100 mL. The antifungal effect of the optimized strain fermentation dilution was significantly enhanced, and the antifungal rate of *R. solani* increased from 27.33 to 59.53%, closely aligning with the predicted value of 53.03%. The results of HPLC-MS/MS and transcriptomic analysis revealed that the content of some secondary metabolic active substances in the fermentation broth of KN37 was significantly different from that of the original fermentation broth. Notably, the content of 4- (diethylamino) salicylaldehyde (DSA) was significantly increased by 16.28-fold while the yield of N- (2,4-dimethylphenyl) formamide (NDMPF) was increased by 6.35 times. Transcriptomic analysis further elucidated molecular mechanisms behind these changes with the expression of salicylic acid dehydrogenase (SALD) was significantly down-regulated, which was only 0.48 times compared to that before optimization. This research successfully optimized the fermentation process of strain KN37 providing a strong foundation for the actual production and application of strain KN37 in agriculture.

## Introduction

Cotton is an economically important crop, and also has high value in the field of agriculture. In the process of cotton production, it is often infested by a variety of plant diseases, such as damping-off, verticillium wilt, boll-splitting disease, etc [1]. Among them, cotton damping-off disease is an important seedling disease caused by *Rhizoctonia solani*, which causes seedling deficiency and ridge breaking in cotton fields [2]. In severely affected areas, the mortality rate of cotton can reach more than 53%, which seriously reduces the cotton yield.

Biological control offers an environment friendly, highly safe and specificity of prevention and control of plant diseases [3]. *Streptomyces* has become the focus of biological control because of its fast growth rate, strong metabolic activity and production of many metabolites. The secondary metabolites from *Streptomyces* are abundant, and 1/3 of the antibiotics found in microorganisms are derived from *Streptomyces* [4]. *Streptomyces* has gained extensive attention in the field of agriculture and is also an important source of microbial pesticides. The secondary metabolites of *Streptomyces* possess a wide range of biological activities including induction, antibiosis, plant growth-promotion effects, and pathogens antagonists [5]. There have been reports on its prevention and treatment of cucumber Fusarium wilt, cotton Verticillium wilt, tomato gray mold and other crop diseases [6], as well as the promotion of crop growth [7]. In order to obtain high-efficiency of biocontrol bacteria, modern fermentation processes and metabolic engineering technology were used to regulate the fermentation process, optimize the fermentation and value-added production process, and improve the yield of effective active substances of biocontrol bacteria. Microbial fermentation mostly adopts liquid fermentation process, and the secondary metabolism level of strains is significantly affected by fermentation conditions.

Response surface methodology is an experimental design method for optimizing and exploring the relationship between response variables and multiple factors. It has been widely used in various industries including pharmaceuticals, food, beverage and agriculture. The Plackett-Burman design (PBD) is a statistical method for rapid screening of significant factors at two levels [8]. The key parameters selected by PBD can be further optimized by central composite design (CCD) and response surface methodology (RSM). RSM is an efficient biological optimization technique that uses a complete quadratic polynomial model to show the relationship between variables. As a part of RSM, CCD is a statistical method that fully considers the interaction of variables and is widely used in optimizing medium conditions [9, 10] and components to improve enzyme production efficiency [11–13]. The optimal fermentation medium and conditions determined by the RSM provide a solid foundation for industrial production after verification.

*Streptomyces* sp. KN37 was isolated from the soil of Kanas, Xinjiang, China in the early stage of our research. Previous studies demonstrated that its fermentation supernatant has a good inhibitory effect on a variety of plant pathogens [14]. The main active secondary metabolites are 4-(diethylamino) salicylaldehyde and 4-nitrosodiphenylamine [15]. In this study, single factor test and PB test were used to screen the influencing factors. To evaluate the antifungal activity, the mycelial growth rate method was used to measure the inhibition of *Rhizoctonia solani* in order to screen the optimal fermentation conditions. High performance liquid chromatography / mass spectrometry (HPLC-MS/MS) was used to detect the difference in the content of secondary metabolites of KN37 strain before and after optimization. Furthermore, we investigated the mechanism of metabolite content change was studied at the transcriptional level. This study aimed to provide theoretical research basis and technical support for the industrial scale fermentation of *Streptomyces*.

## Results

### Determination of optimal carbon and nitrogen source

**Fig. 1:**
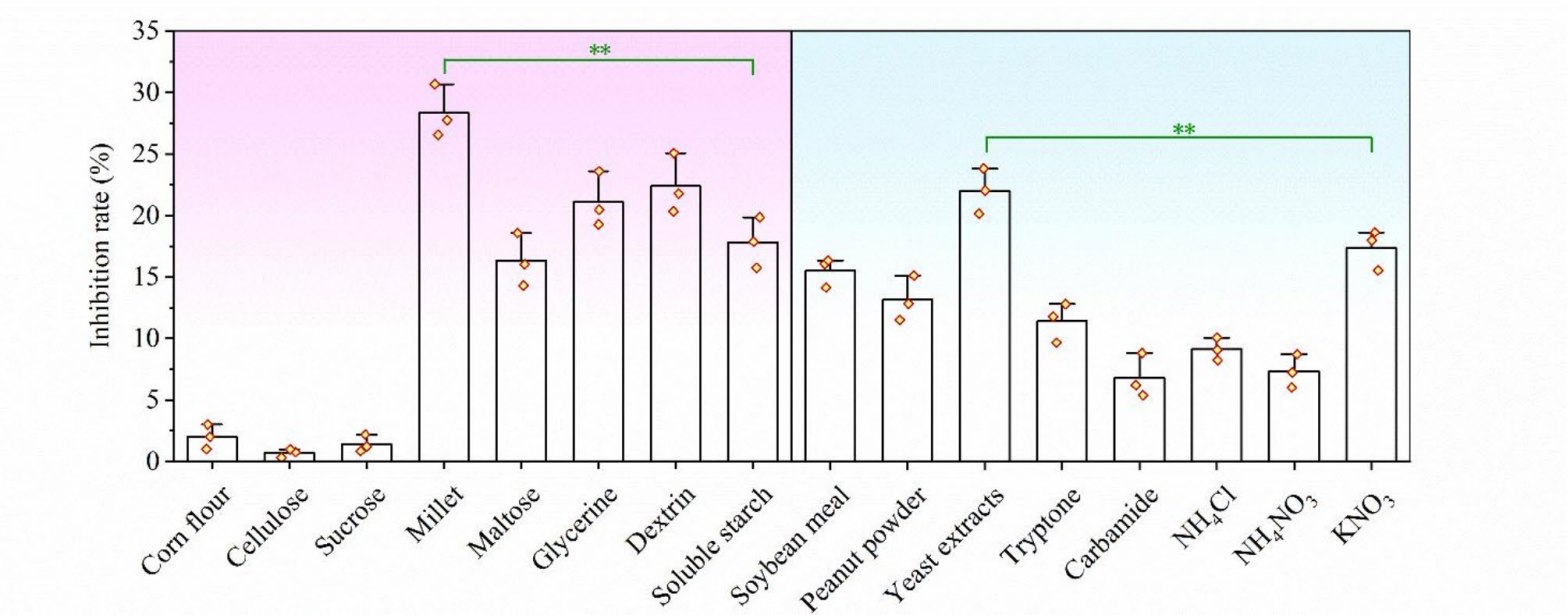
Effects of carbon and nitrogen sources on the biological activity of the diluted fermentation broth of strain KN37. Error bars represent standard deviation. Two asterisks indicate significant differences in the data (*p* < 0.01)

The effect of carbon and nitrogen source on bioactivity of the strain broth were investigated (Fig. 1). The results indicated that using corn starch, cellulose, or sucrose as the sole carbon source significantly reduced the antifungal activity of the strain broth. In contrast, maltose as carbon source did not have a significant impact on bioactivity. The utilization of millet, glycerin or dextrin as the sole carbon source significantly increased the bioactivity. When millet was used as carbon source, the antifungal activity of the broth dilution against *R. solani* was 25% higher than that of the original medium.

Furthermore, replacing KNO_3_ with yeast extracts as the sole nitrogen source significantly enhanced the bioactivity of the strain broth. However, when soybean meal, peanut powder, tryptone, carbamide, NH_4_Cl or NH_4_CO_3_ was used as the sole nitrogen source, the bioactivity was significantly reduced. Based on these findings, millet and yeast extracts were identified as the carbon and nitrogen source of culture medium for further optimization.

### Determination of optimal mineral salt

**Fig. 2:**
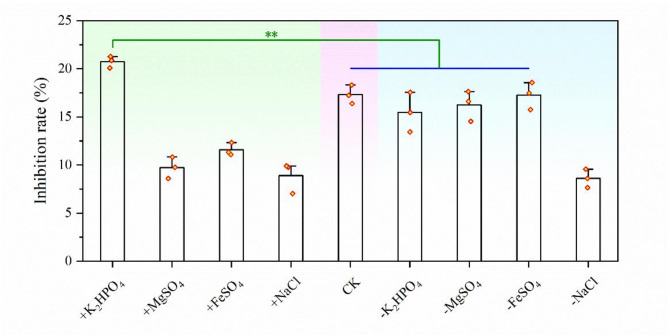
Effect of inorganic salts on bioactivity of the diluted fermentation broth of strain KN37. “+” means that only the inorganic salt is added (forward single factor test), and “-” means that the inorganic salt is removed from the CK (reverse single factor test). Error bars represent standard deviation. Two asterisks indicate significant differences in the data (*p* < 0.01)

A two-way single-factor experimental method was used to further optimize GOM basal medium (Fig. 2). The results of forward single-factor experiment indicated that the bioactivity of the treatment with the addition of K_2_HPO_4_ was greater than that of the control. Conversely, in the results of reverse single-factor experiment, there was no significant difference in the bioactivity between the treatment groups without the addition of K_2_HPO_4_, MgSO_4_, or FeSO_4_ and the control group. The two-way single-factors experiment indicated that the addition of K_2_HPO_4_ contributed to a notable improvement in antifungal activity. However, the treatment group with the addition of MgSO_4_, FeSO_4_ or NaCl showed a lower inhibition rate than the control group, and there was no significant difference between the treatment group without the addition of MgSO_4_ or FeSO_4_ and the control group. Therefore, K_2_HPO_4_ was selected as the optimal mineral salt in the culture medium.

### Determination of optimal culture conditions

**Fig. 3:**
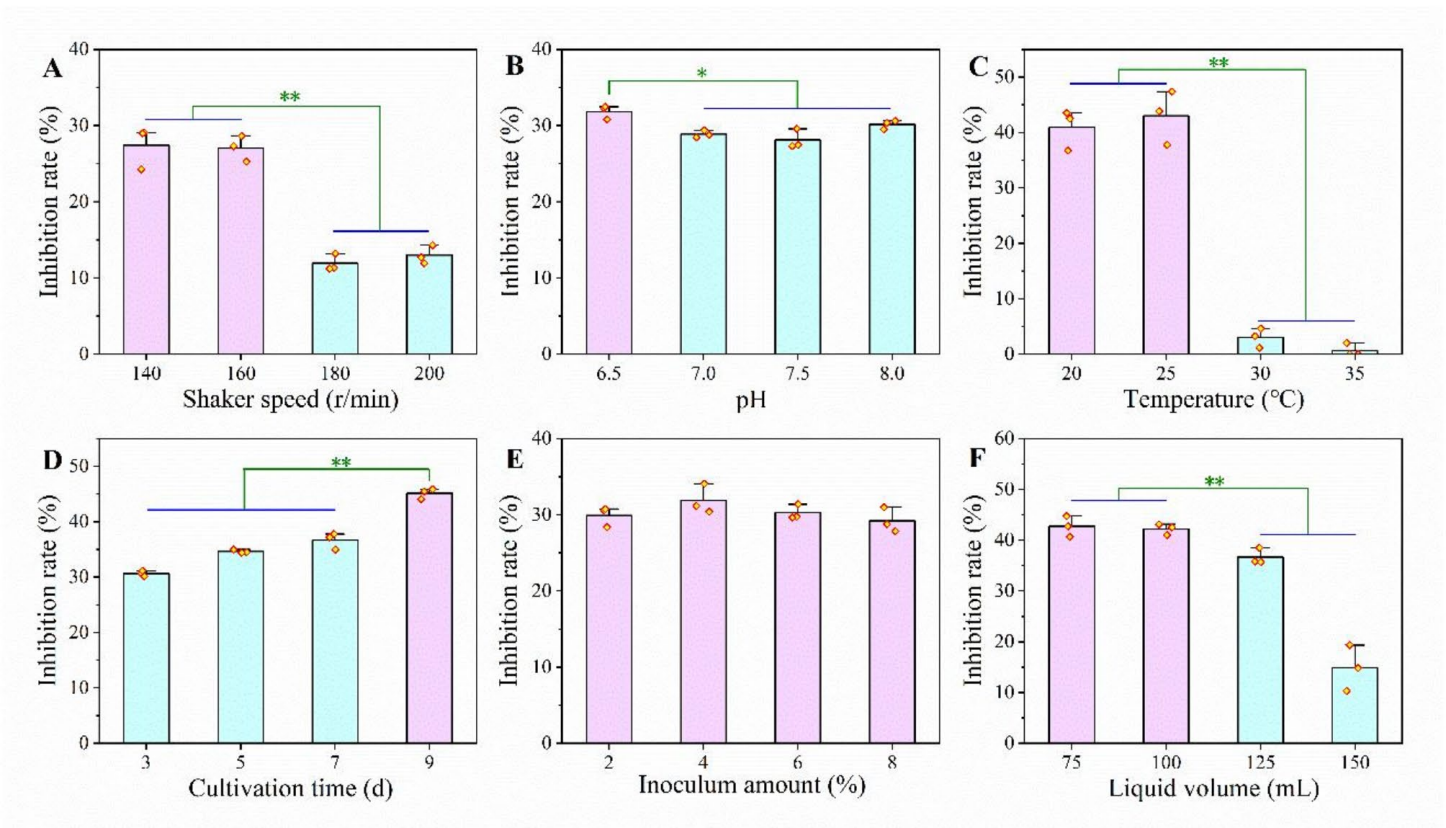
Effects of different culture conditions on bioactivity of the diluted fermentation broth of strain KN37. Error bars represent standard deviation. Two asterisks indicate significant differences in the data (*p* < 0.01)

The effects of fermentation conditions on the antifungal activity of the broth dilution are shown in Fig. 3. The effects of rotation speed, initial pH, temperature, fermentation time, inoculation amount and liquid volume on the antifungal activity of the broth dilution were evaluated. The results showed that the broth dilution had the highest antifungal activity (Fig. 3A) at the stirring speed of 140 and 160 r/min, and the antifungal rates were 37.51% and 37.32%, respectively. However, with the further increase of stirring speed, the antifungal activity of the fermentation dilution decreased significantly. The fermentation dilution showed the strongest antifungal activity at the initial pH of 6.5, and the inhibition rate was 31.67% (Fig. 3B). With the increase of initial pH value, the antifungal activity of the broth dilution decreased slightly. Secondly, the fermentation dilution reached the highest antifungal activity at 25 °C, reaching 41.39% (Fig. 3C). Subsequently, with the increase in temperature, the antifungal activity of the fermentation dilution decreased significantly. When the fermentation time was 9 days, the fermentation dilution showed the strongest antifungal activity, reaching 44.93% (Fig. 3D). With the increase of fermentation time, the antifungal activity of broth dilution increased continuously. According to Fig. 3E, it was found that with the increase of inoculation amount, the antifungal activity first showed an increasing trend and then decreasing. When the inoculation amount was 4%, the antifungal activity of actinomycetes KN37 broth dilution reached the maximum of 32.1%. Figure 3F showed that when the liquid volume was 75 mL, the antifungal activity of the fermentation dilution was the strongest, which was 42.5%, followed by 42.33% when the liquid volume was 100 mL. However, with the increase in liquid volume, the activity decreased significantly. In summary, the optimal fermentation conditions were as follows: the rotation speed was 140 r/min, the temperature was 25 °C, the initial pH was 6.5, the culture time was 9 days, the inoculation amount was 4%, and the liquid volume was 100 mL. These conditions yielded the highest antifungal activity.

### PBD experiments to screen the main effect factors on bioactivity

**Fig. 4:**
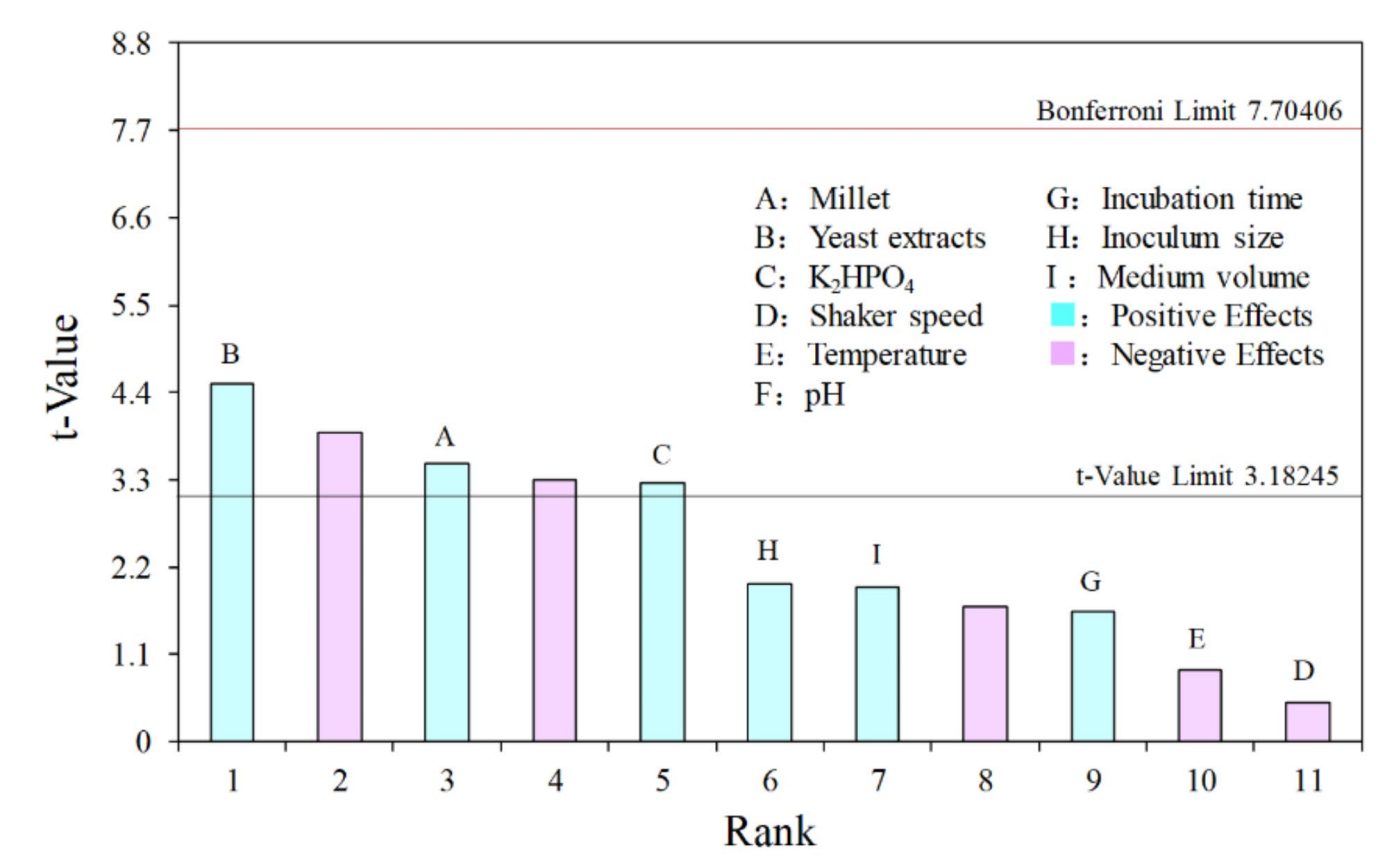
The Pareto chart of PBD experiments

The PBD (Plackett-Burman Design) was conducted using Design-Expert software to identify the main factors that have a significant impact on the biological activity of the strain broth (Table 1). Based on the Pareto chart (Fig. 4), millet, yeast extract, and K_2_HPO_4_ were identified as the top three factors with the highest impact (*t*-value > 3.18). Furthermore, these three medium component factors exhibited positive effects on the antifungal activity.

**Table 1:** Result of PBD experiment

Run	Coded level of factors									Inhibition rate (%)
	A	B	C	D	E	F	G	H	I	
1	1	1	1	-1	-1	-1	1	-1	1	23.61
2	-1	1	1	-1	1	1	1	-1	-1	27.6
3	1	-1	1	1	-1	1	1	1	-1	21.12
4	-1	-1	-1	1	-1	1	1	-1	1	7.56
5	-1	1	1	1	-1	-1	-1	1	-1	30.11
6	-1	-1	-1	-1	-1	-1	-1	-1	-1	15.53
7	1	-1	-1	-1	1	-1	1	1	-1	4.5
8	-1	1	-1	1	1	-1	1	1	1	28.44
9	1	1	-1	-1	-1	1	-1	1	1	16.56
10	1	1	-1	1	1	1	-1	-1	-1	29.68
11	1	-1	1	1	1	-1	-1	-1	1	0.99
12	-1	-1	1	-1	1	1	-1	1	1	29.66

### CCD experiments to screen the optimal culture conditions

Design-Expert software was used for CCD and results analysis, with millet, yeast extract and K_2_HPO_4_ as experimental factors, the biological activity of the broth dilution as the response value, and three-factor, three-level optimization experiments were performed, and the specific experimental design and results are shown in Table 2.

**Table 2:** Result of CCD experiment

Run	Coded level of factors			Inhibition rate (%)	
	A	B	C	Observed	Predicted
1	0	0	0	36.26	36.60
2	1	-1	1	37.22	37.50
3	0	0	-1.682	43.82	44.31
4	0	0	0	38.55	36.60
5	0	0	0	35.5	36.60
6	1	1	-1	40.34	40.61
7	1	-1	-1	53.81	53.03
8	1.682	0	0	30.9	31.27
9	-1	-1	1	37.63	36.26
10	-1	1	-1	42.85	41.47
11	0	1.682	0	45.44	46.51
12	0	0	0	40.54	36.60
13	-1.682	0	0	29.76	30.95
14	0	-1	0	56.63	57.11
15	-1	1	1	36.39	36.07
16	-1	-1	-1	44.23	44.41
17	1	1	1	29.11	27.83
18	0	0	0	33.44	36.60
19	0	0	1.682	25.64	26.71
20	0	0	0	35.58	36.60

The regression equation was obtained as: Y = 36.60 + 0.095 A − 3.15B – 5.23 C – 2.37AB − 1.84AC + 0.69BC − 1.94A^2^ + 5.38B^2^ − 0.39C^2^. Where Y is the inhibition rate (%), A, B, and C are millet, yeast extract and K_2_HPO_4_, respectively.

The regression model was analyzed and the results are shown in Table 3. Item C and item B^2^ had a highly significant effect on Y values (*p* < 0.0001), term B, term AB, term AC and term A^2^ had a significant effect on Y values (*p* < 0.05), and the remaining terms did not have significant effects. The model *p* < 0.0001, indicating that the regression is significant, and the misfit term *p* = 0.8724 > 0.05, indicating that the misfit is not significant, and the model *R*^2^ = 0.9629, indicating that 96.29% of the experimental results can be explained using the model, and *R*^2^ adj = 0.9296, indicating that the actual value is also close to the predicted value of the model, therefore, the combination indicates that the model has high credibility that can be used to predict the biological activity of the broth dilution.

**Table 3:** ANOVA for response surface quadratic model

Source	Sum of squares	df	Mean square	F value	*p*-value prob > F	
Model	1098.62	9	122.07	28.88	< 0.0001	significant
A-Millet	0.12	1	0.12	0.029	0.8678	
B-Yeast extracts	135.51	1	135.51	32.06	0.0002	
C-K_2_HPO_4_	373.86	1	373.86	88.44	< 0.0001	
AB	44.94	1	44.94	10.63	0.0086	
AC	27.23	1	27.23	6.44	0.0295	
BC	3.78	1	3.78	0.89	0.3665	
A^2^	54.38	1	54.38	12.87	0.0050	
B^2^	416.77	1	416.77	98.59	< 0.0001	
C^2^	2.16	1	2.16	0.51	0.4912	
Residual	42.27	10	4.23			
Lack of Fit	10.61	5	2.12	0.33	0.8724	not significant
Pure Error	31.67	5	6.33			
Cor. Total	1140.90	19				

Based on the regression model, a three-dimensional response surface of the activity of the broth dilution was plotted, visualizing the impact of independent variables on the response values. The effects of the other two variables on the response values were compared by keeping the third variable at its zero level. A steeper the slope and more pronounced curvature of the response surface indicate the greater impact of the factors on the response values.

**Fig. 5:**
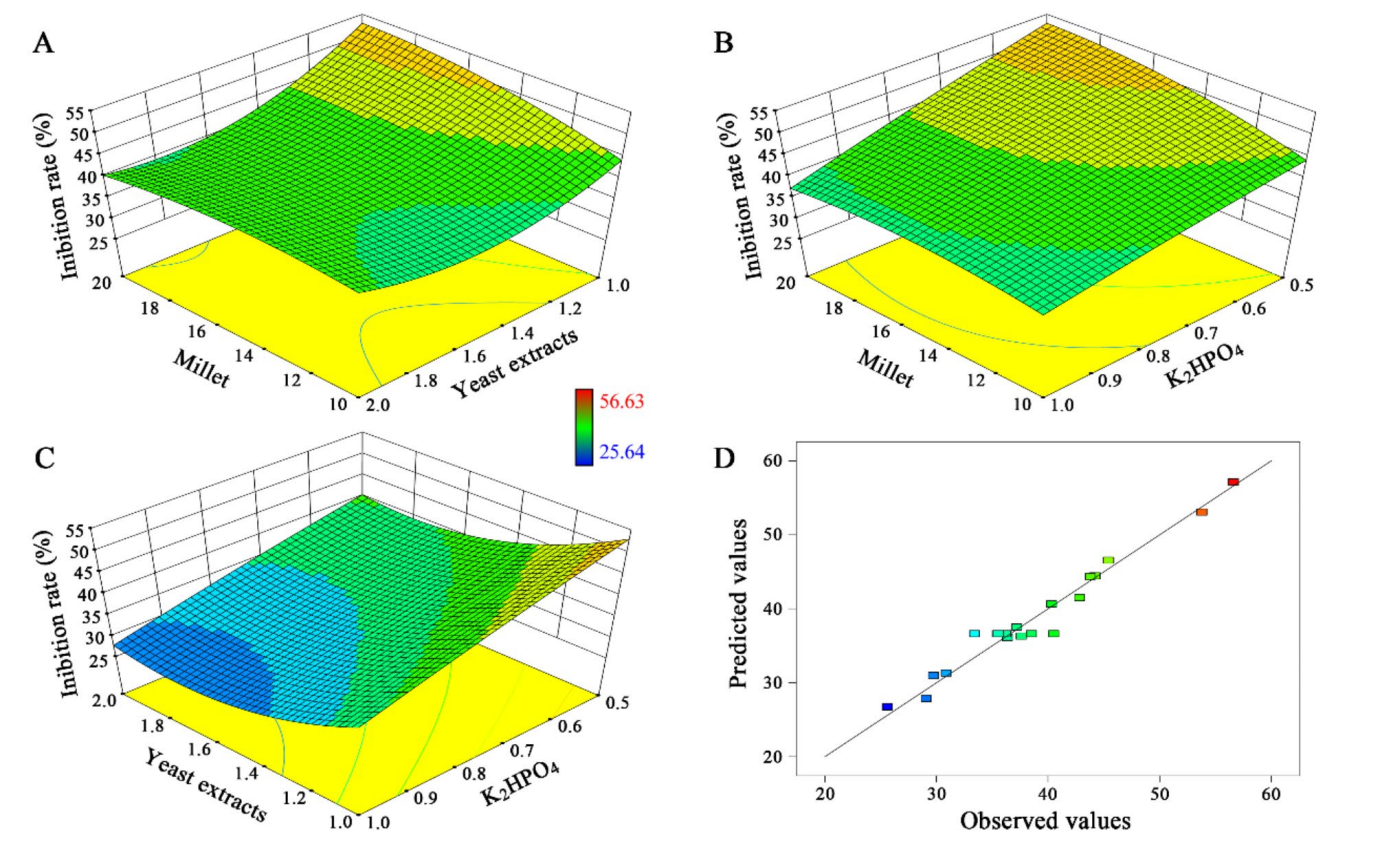
Fitting response surface analysis of inhibition rate, predicted value and measured value of actinomyces KN37

From the response surface analysis, it can be observed that in the interaction plot of yeast extract addition and millet addition in the medium (Fig. 5A), the curvature of yeast extract addition is greater than that of millet addition, indicating that yeast extract addition has a greater impact on the bioactivity than millet addition. In the interaction plot of millet addition and K_2_HPO_4_ addition in the medium (Fig. 5B), the curvature of millet addition is greater than that of K_2_HPO_4_ addition which indicate that millet addition has a greater impact on the activity of the broth dilution than K_2_HPO_4_ addition. Similarly, in the interaction plot of yeast extract addition and K_2_HPO_4_ (Fig. 5C), the curvature of yeast extract addition is greater than that of K_2_HPO_4_ indicating that yeast extract has a greater impact on the activity of the broth dilution than K_2_HPO_4_. In summary, the three factors had significant effects on the biological activity of fermentation solution in the following order: yeast extract> millet> K_2_HPO_4_. This ranking is consistent with PB experiment results. The relationship between the predicted response and the experimental results showed that almost all the predicted values are basically in agreement with the observed values (Fig. 5D).

Using Design-Expert V8.0.6 software, the optimal fermentation conditions were determined to be: 20 g/L of millet, 1 g/L of yeast extract, and 0.5 g/L of K_2_HPO_4_, at which the inhibition rate of the fermentation dilution reaches the theoretical maximum of 53.03%.

### Model validation

**Fig. 6:**
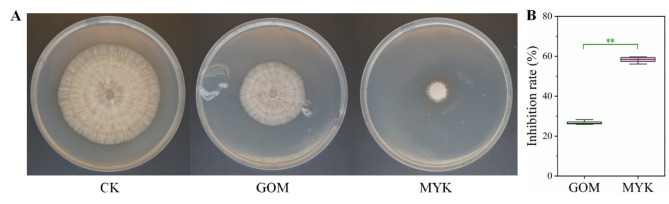
Difference of bioactivity between GOM and MYK medium. **A**: colony difference, **B**: inhibition rate difference. Error bars represent standard deviation. Two asterisks indicate significant differences in the data (*p* < 0.01)

In order to determine whether the model prediction results are consistent with the actual measurement results, the optimized medium (MYK medium: millet 20 g, yeast extract 1 g, K_2_HPO_4_•3H_2_O 0.5 g, pure water 1000 mL, pH 6.5) and the original GOM medium were used for a three-part comparative verification test. The inhibitory effects of the fermentation dilution of the strain on *R. solani* before and after optimization are shown in Fig. 6. After optimization, the colony growth treated with KN37 strain’s fermentation dilution was significantly inhibited, with the inhibition rate increasing from 27.33 to 59.53%, which was in good agreement with the predicted value of 53.03%.

**Fig. 7:**
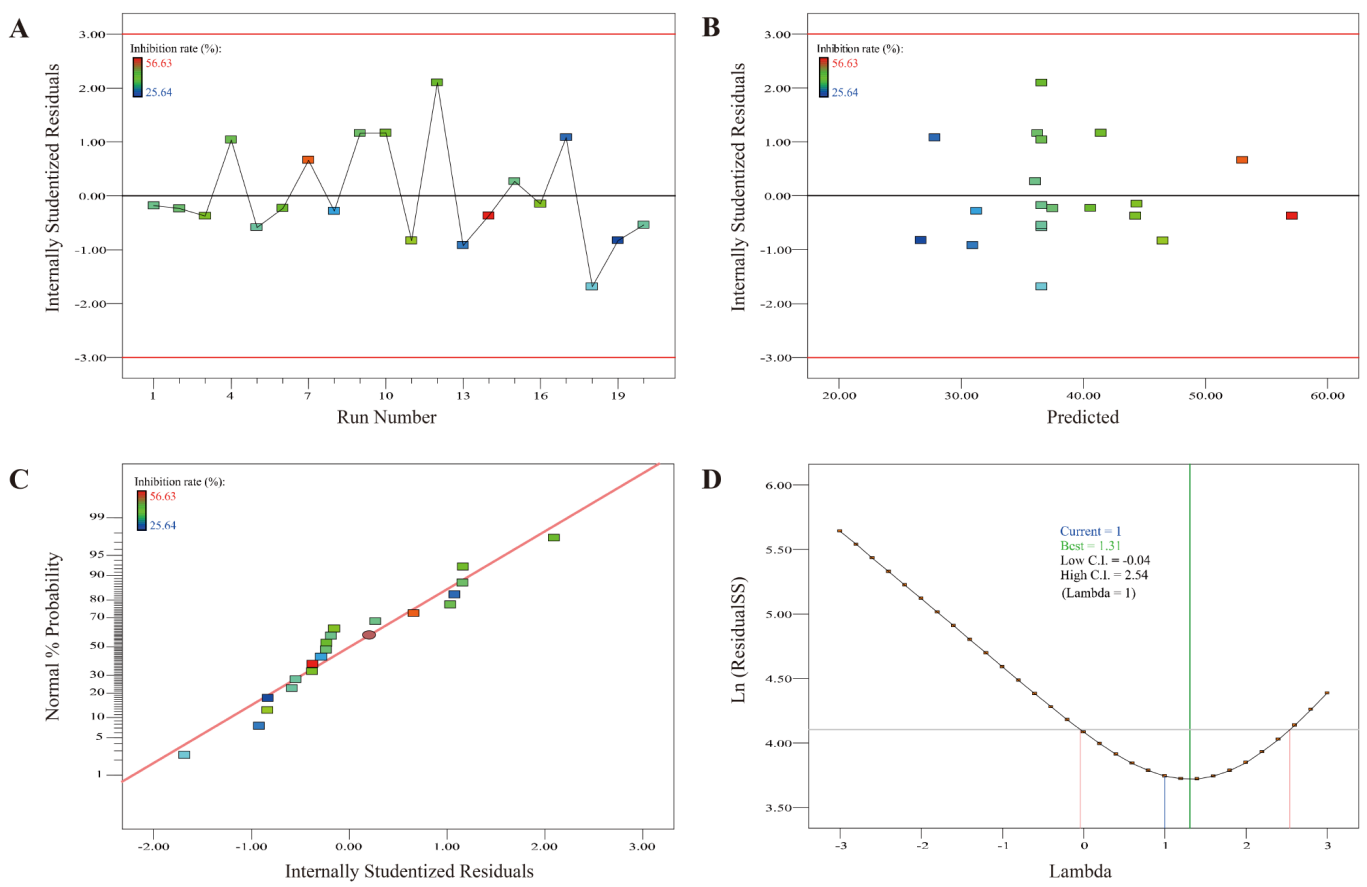
The residual versus run order (**A**), Scatter plot of studentized residual and predicted value (**B**), Normal probability diagram of residuals(**C**) and Box-Cox diagram (**D**) of the data based on CCD experimental design

We also created the residual diagram, running diagram, and Box-Cox diagram for the data from the CCD experimental design. The results indicate that the running chart shows the residuals fluctuating randomly around the zero line, suggesting that the residuals are independent and that the model is appropriate (Fig. 7A). The residual graph indicates the residuals are randomly distributed and fluctuate near the 0 line, suggesting that the model fits well (Fig. 7B and C). The Box-Cox diagram suggests that the data is close to a normal distribution and may not need to be transformed (Fig. 7D).

### Metabolome and transcriptome analysis

**Fig. 8:**
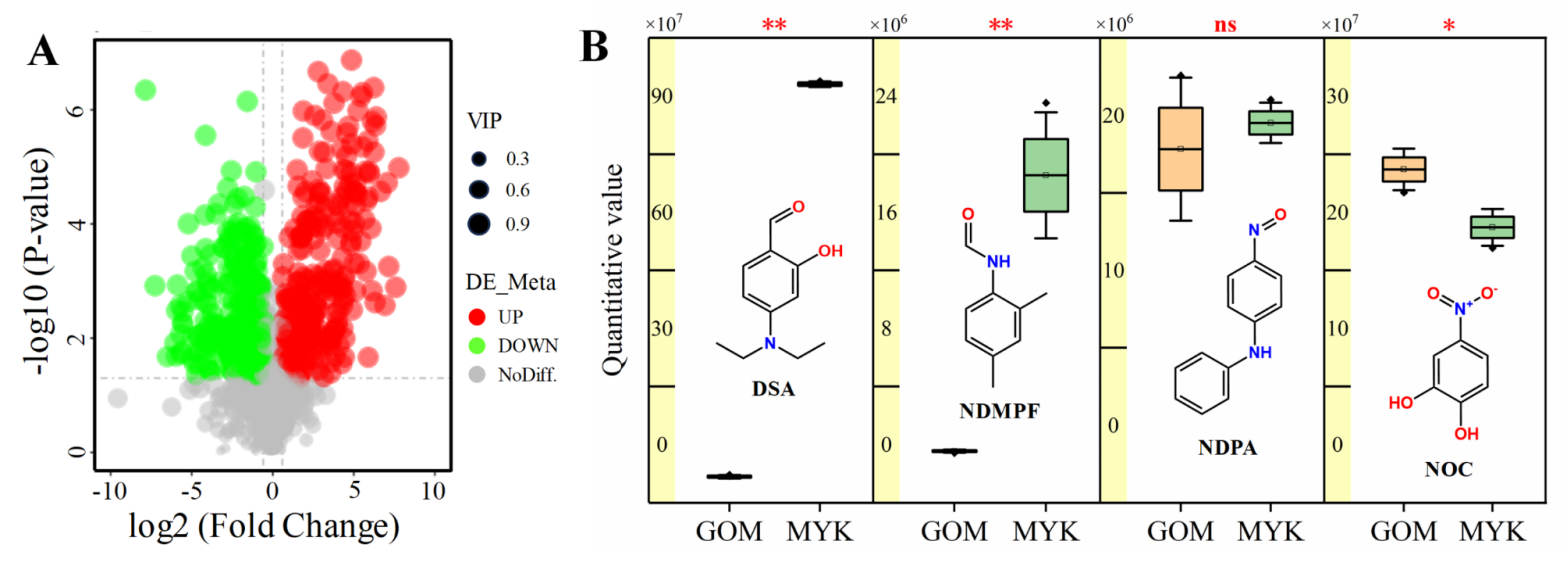
Metabolic differences of strains cultured in GOM and MYK medium. **A**: Volcano plot of metabolomics differences, **B**: Differential content of DSA in metabolites. Two asterisks indicate significant differences in the data at the *p* < 0.01 level. Single asterisk indicates significant differences in the data at the *p* < 0.05 level. “ns” indicates that there is no significant difference in the data

In order to clarify the changes in the content of secondary metabolites in the broth dilution of strain KN37 cultured in the medium before and after optimization, we conducted HPLC-MS analysis on the broth dilution supernatant before and after optimization. After MYK cultivation, HPLC-MS analysis revealed that the levels of 267 metabolites in the strains significantly increased, while 252 metabolites decreased (Fig. 8A). Specifically, the content of 4-(Diethylamino) salicylaldehyde (DSA) notably increased, with the content in MYK culture medium being 16.28 times that of GOM. The yield of N- (2,4-Dimethylphenyl) formamide (NDMPF) increased by 6.35 times, the yield of 4-Nitrosodiphenylamine (NDPA) did not change, and the yield of 4-Nitrocatechol (NOC) decreased slightly by 17.37% (Fig. 8B). Our previous experiments showed that DSA and NDMPF had a significant inhibitory effect on *R. solani* [15]. Therefore, the content of DSA and NDMPF in the optimized KN37 broth dilution increased significantly, which improved the antifungal activity. Further investigation is required to study the mechanisms responsible for the accumulation of secondary metabolites.

**Fig. 9:**
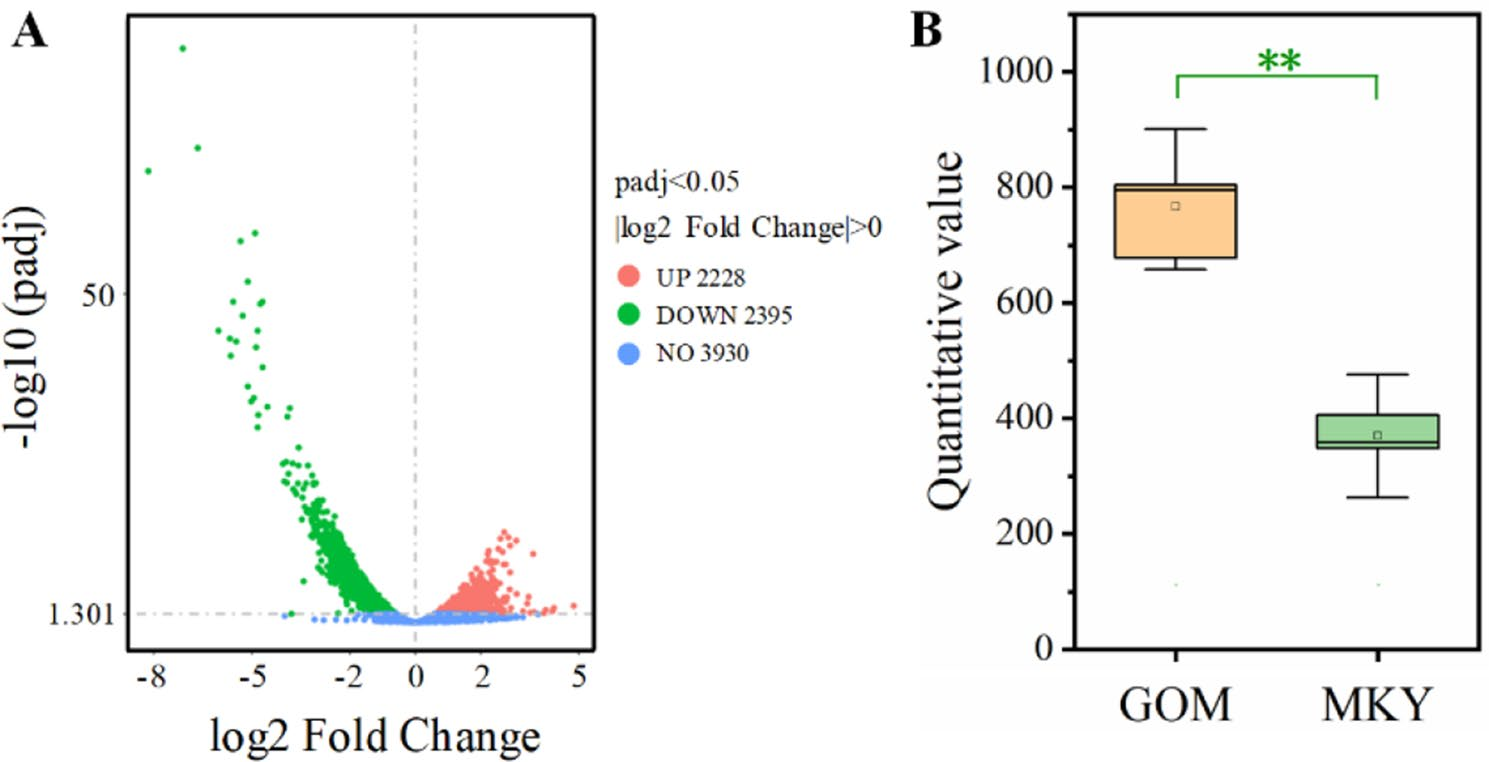
Transcriptomic differential analysis of strains cultured in GOM and MYK medium. **A**: Volcano plot of transcriptomic differences, **B**: Expression of salicylaldehyde dehydrogenases in strains cultured in different medium. Two asterisks indicate significant differences in the data (*p* < 0.01)

Transcriptomic analysis results indicated that in MYK 2228 genes were upregulated and 2395 genes were downregulated (Fig. 9A). Notably, the expression of salicylaldehyde dehydrogenase (SALD) was significantly downregulated, with its expression in MYK being only 0.48 times that of GOM (Fig. 9B).

**Fig. 10:**
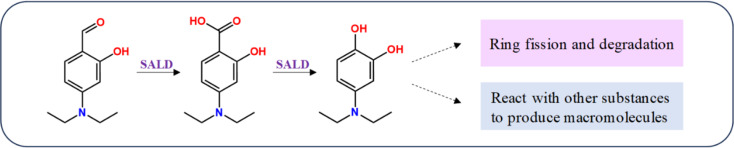
The proposed catabolic pathways of DSA in strain KN37

Salicylaldehyde dehydrogenase (SALD) catalyzed the last reaction in the upper pathway of naphthalene degradation: the oxidation of salicylaldehyde to salicylate [16]. DSA is a small molecule metabolite of actinomycete KN37 and also an important intermediate in its metabolic biosynthesis process. It can be hydrolyzed to 4-(diethylamino) salicylic acid by SALD, and subsequently transformed into 4-(diethylamino) phenol which can further degrade to pyruvic acid entering the tricarboxylic acid cycle, or react with other substances to form macromolecules (Fig. 10). The reduction of SALD leads to a significant accumulation of DSA, thereby resulting in a remarkable enhancement of the antifungal activity of the broth dilution.

## Discussion

Actinomycetes produce new secondary metabolites mainly depending on the strain, nutritional components in the fermentation medium and the fermentation conditions [17]. The secondary metabolites (antibiotics) synthesized by actinomycetes vary greatly with different culture media or culture conditions. Changes in culture media components or culture conditions may lead to a complete loss or increase in antibiotic production [18].

The present study identified that millet is the most suitable carbon source for the growth and reproduction of strain KN37. These findings are consistent with those of Zhou et al. [19]. Millet, as a carbon source, is more conducive to the production of active substances in the broth dilution, possibly because millet is rich in protein, fat, and vitamin B, which can provide more abundant nutrition for the growth of actinomycetes. Yeast extract was found to be the most suitable nitrogen source and is an essential element in nucleic acid and protein synthesis as well as plays a crucial role in the growth and development of microorganisms [20]. When used as a nitrogen source, yeast extract exhibits stronger antifungal activity compared to KNO_3_, these results were consistent with the findings of Liu et al. [21]. This suggests that yeast extract is more suitable for the growth and reproduction of the strain, because it is rich in amino acids and vitamin B, which support the production of secondary metabolites by the strain.

When K_2_HPO_4_ was added individually to the culture medium, strain KN37 exhibited the highest antifungal activity in the fermentation dilution. Research by Kiranmayi, et al. found that K_2_HPO_4_ promotes the production of cellular biomass and biologically active metabolites [22]. However, in the study by Wang, et al., the addition of K_2_HPO_4_ decreased biological activity [23]. This indicates that different strains have varying requirements for metal ions.

The fermentation conditions also significantly impact the fermentation culture of the strain. The optimal culture conditions obtained in this experiment using the single-factor method were: rotation speed at 140 r/min, temperature at 25 ℃, initial pH of 6.5, culture time of 9 days, inoculum size of 4%, and liquid volume of 100 mL. Different types of microorganisms have distinct optimal growth pH levels [24]. Even for the same type of microorganism, their demand for the optimal pH may vary in different growth stages and different physiological and biochemical activities [25, 26]: a high inoculum size may lead to excessive accumulation of metabolic products, causing a stress response; while a low inoculum size may lead to a delay in the fermentation cycle [27]. A high liquid volume will affect the aeration, thereby impacting the accumulation of active substances. The higher the rotation speed, the larger the bacteria, which also has a certain effect on the dissolved oxygen level [26]. The strain exhibits better antifungal activity in relatively low-temperature environments, whereas high temperatures significantly inhibit its activity, which may be related to the strain’s own growth environment [28].

In general, the antimicrobial effect of biocontrol bacteria against plant pathogens depends on the amount of specific bioactivity substances or the size of the inhibitory zone [25, 29]. In this study, we compared the levels of the known KN37 metabolites before and after optimization, and concluded that the yield of these four antifungal substances was not proportional to the results of culture optimization. This indicates that the content of a specific antifungal substance cannot be used alone as a response variable to optimize the culture conditions. Instead, we used the inhibition rate of the broth dilution on the mycelial growth of pathogenic fungi as an optimized response variable.

To understand the interaction between the key factors obtained from single factor experiments, we use statistical design methods to optimize the fermentation process. PBD can screen out the most important variables affecting biological activity [30, 31]. RSM was used to optimize fermentation conditions to meet the nutritional needs of specific microorganisms, thereby avoiding unnecessary addition of excessive components in the medium [26]. The fermentation conditions and medium of the optimal activity were screened by the response surface model. The model verification results showed that the mycelial inhibition rate of strain KN37 against *R. solani* increased from 27.33 to 59.53%, which was in good agreement with the predicted value of 53.03%. The antifungal activity of the broth dilution of the strain KN37 was significantly improved by fermentation optimization, and the reasons were elucidated by HPLC-MS analysis: (1) The optimization of fermentation conditions increased the yield of active antifungal components; (2) The metabolic profile of the strain changed, resulting in more active antifungal components. The reason for the increase of 4- (Diethylamino) salicylaldehyde (DSA) production is the continuous accumulation of DSA caused by the significant down-regulation of salicylaldehyde dehydrogenase (SALD) expression.

In this study, the development, evaluation and optimization of fermentation characteristics of strain KN37 were carried out to improve the fungicidal activity and biological control effect. To the best of our knowledge, this is the first time the Plackett-Burman model and RSM have been used to evaluate and enhance the fermentation characteristics and the antifungal efficacy of strain KN37 in the fermentation process. The potential reasons for the significant increase in the activity of the optimized fermentation broth were fully elucidated through the metabolomic and transcriptomic analysis. The optimization model developed in this study establishes a more effective and efficient fermentation process, potentially paving a way to develop novel microbial based biological control agent in the future.

## Conclusions

The present study reported the first comprehensive analysis of the optimal medium formula and fermentation conditions of *Streptomyces* sp. KN37 elucidating the underlying mechanisms for the improvement of antifungal activity of the fermentation broth of strain KN37 at both the transcriptomic and the metabolomic levels, providing a valuable data for the industrial production of KN37. Based on the results of RSM and PB design we effectively identified the ideal medium and fermentation parameters. The combination of metabolic and transcriptomic analysis offered novel insights into the mechanism of its activity improvement. Notably, this study first obtained the content change of strain KN37 active secondary metabolites from the metabolic level, and then investigated the reasons for the activity change at transcriptomic level, thus fully explaining the effect of changes in culture conditions on the activity of the strain. Based on the above results, the optimization of the fermentation medium composition and fermentation conditions of strain KN37 provides a theoretical basis for the actual field application and industrialization, and also highlights the necessity for further exploration into the antifungal mechanisms of the active substances contained in the fermentation broth.

## Materials and methods

### Microorganism strain and chemicals

*Streptomyces* sp. KN37 (CGMCC 13160) was previously isolated from Kanas Lake of China and was preserved in our laboratory. *R. solani* was isolated from diseased cotton seedlings. The isolated strain was suspended in 20% glycerol and stored at − 80 °C until further use. All chemicals used in the research were obtained from Solarbio Science & Technology Co., Ltd., China.

### Medium and culture condition

The basal liquid medium used in the study was Gao’s No.1 medium (GOM) which consisted of: soluble starch 20 g, KNO_3_ 1 g, MgSO_4_·7H_2_O 0.5 g, NaCl 0.5 g, K_2_HPO_4_ 0.5 g, ferrous sulfate 0.01 g, pH 7.3–7.5.

Preparation of seed culture: The activated strain was punched with a 5 mm puncher and inoculated into a 250 mL conical flask containing 100 mL GOM. Eight bacterial cakes were inoculated per 100 mL, and cultured at 28 °C and 180 r/min for 3 days.

Fermentation broth preparation: In a 250 mL shake flask containing 100 mL fermentation medium, the seed culture was inoculated at a ratio of 4% (v/v). The fermentation process was carried out at 28 °C with shaking at 160 r/min for 7 days. The fermentation broth was diluted 10 times with sterile water to make broth dilution, and then passed through a 0.22 μm bacterial filter and stored at 4 °C for further use.

### Determination of biological activity

The mycelial growth rate method was used to determine the biological activity of the broth dilution [32]. The broth dilution was mixed with PDA medium at a ratio of 1:9 to make a drug-carrying medium while the blank control consisted of PDA medium which was added with the same amount of sterile water. A 5 mm *R. solani* fungus cake was inoculated on the both the drug-carrying medium and the blank medium, followed by incubation at 28 °C for 2–3 days in dark. Each treatment was replicated three times. The colony diameter was measured by the cross-crossing method and the inhibition rate was calculated by the following formula.

$$\begin{aligned}{\rm{Inhibition rate \,(\%)}} =&\\ &{\matrix{{\rm{}} \hfill \cr {\rm{control colony diameter - treatment colony diameter }} \hfill \cr} \over {{\rm{control colony diameter }}}}\, \times {\rm{ }}100{\rm{ }}\%\end{aligned}$$

### Single factor experiment screened the best carbon source

On the basis of GOM liquid medium formulation, as detailed in the Sect. 2.2 for broth dilution, 20 g/L soluble starch was replaced by 20 g/L corn flour, cellulose, sucrose, millet flour, maltose, glycerol and dextrin to screen the carbon source in the medium, and other components remained unchanged. GOM liquid medium without carbon source was used as control. Each treatment was repeated 3 times. After fermentation, the biological activity of the broth dilution was determined according to the determination method detailed in Sect. 2.3.

### Single factor experiment screened the best nitrogen source

On the basis of GOM liquid medium formulation, according to the preparation method given in Sect. 2.2 for broth dilution, 1 g/L KNO_3_ was replaced by 1 g/L soybean meal, peanut powder, yeast extract, tryptone, urea, NH_4_Cl and ammonium bicarbonate to screen the nitrogen source in the medium, and the other components remained unchanged. GOM liquid medium without nitrogen source was used as control. Each treatment was repeated 3 times. After fermentation, the biological activity of the broth dilution was determined according to the determination method of 2.3.

### Forward single-factor experiment screened the best mineral salt

The soluble starch and KNO_3_ were used as carbon and nitrogen sources, and K_2_HPO_4_ (0.5 g/L), MgSO_4_•7H_2_O (0.5 g/L), FeSO_4_•7H2O (0.01 g/L), NaCl (0.5 g/L) were each added into medium as the only mineral salt, respectively. Another group of experiments, on the basis of GOM, K_2_HPO_4_, MgSO_4_•7H_2_O, FeSO_4_•7H_2_O and NaCl were each subtracted, successively. GOM liquid medium was used as the control group. Each treatment was repeated 3 times. According to the preparation method of broth dilution described in 2.2. After fermentation, the biological activity of the broth dilution was determined according to the method described in Sect. 2.3.

### Optimization of fermentation culture conditions

Using the optimal medium formulation selected from the above single factor experiments, the effects of different initial pH values, fermentation temperature, fermentation time, inoculation amount, liquid volume and fermentation speed on the antifungal activity of strain KN37 broth dilution were studied by single factor experiment and single variable principle. The initial pH value of fermentation (6.5, 7.0, 7.5, 8.0), fermentation temperature (20, 25, 30, 35 °C), fermentation time (3, 5, 7, 9 days), inoculation volume (2, 4, 6, 8%), liquid volume (75, 100, 125, 150 mL), and fermentation speed (140, 160, 180, 200 r/min) were optimized, with each treatment being repeated three times. Broth dilution**s** were prepared as described in the Sect. 2.2 and after fermentation, the inhibitory activity of the broth dilution**s** against *R. solani* was determined by the mycelial growth rate method.

### Plackett-Burman design experiments

Based on the results of each component in the medium, the Plackett-Burman design (PBD) was used to determine the key factors of the medium components: carbon source (millet), nitrogen source (yeast extracts), mineral salt (K_2_HPO_4_) and culture conditions (shaker speed, temperature, pH, time, inoculum size and medium volume). Broth dilutions were prepared as described in the Sect. 2.2 and after fermentation, the biological activity of the broth dilution was determined. Design-Expert software was used to perform the PBD for *n* = 9, and the factors and levels of the PBD are shown in Table 4.

**Table 4:** The factors and levels in PBD experiments

Code	Factor	Level		
		-1	0	-1
A	Mellit (g/L)	15	20	25
B	Yeast extracts (g/L)	0.5	1	1.5
C	K_2_HPO_4_ (g/L)	0.25	0.5	0.75
D	Shaker speed (r/min)	120	140	160
E	Temperature (℃)	20	25	30
F	pH	6	7	8
G	Time (d)	5	7	9
H	Inoculum size (%)	2	4	6
I	Medium volume (mL)	50	100	150

### Central composite design experiments

The Central Composite Design (CCD) was used to further evaluate the screened principal component factors (millet, yeast extracts, K_2_HPO_4_). The levels of the principal component factors were independent variables, and each variable has 5 levels. The bioactivity of broth dilution was determined after fermentation. The data from the CCD experimental design is processed using Design-Expert software, and the Box-Cox diagram is exported to confirm that no transformation is required. The residual and running diagrams are used to ensure that the residuals do not exhibit any trends during continuous operation. The factors and levels in the experimental protocol are shown in Table 5.

**Table 5:** The factors and levels in CCD experiments

Code	Factor	Level				
		-α	-1	0	+ 1	+α
A	Mellit (g/L)	6.6	10	15	20	23.4
B	Yeast extracts (g/L)	0.66	1	1.5	2	2.34
C	K_2_HPO_4_ (g/L)	0.33	0.5	0.75	1	1.17

### Model validation

The optimal fermentation conditions obtained from response surface analysis were used for fermentation. The antifungal activity of strain KN37 broth dilution under the fermentation conditions before and after optimization was compared. Broth dilution**s** were prepared as described in the Sect. 2.2 and following the fermentation, the biological activity of the broth dilution was determined according to the determination method given in the Sect. 2.3.

### HPLC-MS/MS analysis

The strain KN37 was cultured in the both optimized medium (MYK medium) and the original medium (GOM medium) for 7 days. A 1 mL of fermentation broth sample was freeze-dried in a freeze-drying machine, and 100 µL of 80% methanol aqueous solution was added. The mixture was left to stand on ice bath standing for 5 min, 15,000 g, 4 °C centrifuged for 15 min. The resulted supernatant was diluted with mass spectrometry grade water to a methanol content of 53%; centrifuged 15,000 g at 4 °C for 15 min, the supernatant was collected, and analyzed using UHPLC-MS/MS for analysis [33].

HPLC-MS / MS analysis was performed using a Vanquish UHPLC system (Thermo Fisher, Germany) coupled with an Orbitrap Q ExactiveTM HF-X mass spectrometer (Thermo Fisher, Germany) Q Exactive TM HF / Q ExactiveTM. The samples were injected into Hypesil Gold column (100 × 2.1 mm, 1.9 μm) with a linear gradient of 12 min at a flow rate of 0.2 mL/min. The eluents of positive and negative modes were: positive mode A (0.1% formic acid) and B (methanol); negative mode: A (5 mM ammonium acetate, pH 9.0) and B (methanol). The solvent gradient was set as: 2% B, 1.5 min; 2–85% B, 3 min; 85 ~ 100% B, 10 min.

### Transcriptomic analysis

The strain KN37 was cultured and fermented on GOM medium and MYK medium respectively for 7 days. The cells were collected, frozen in liquid nitrogen and stored at -80 °C. Three repetitions were set respectively. The bacterial samples were sent to Novogene (Beijing, China) for transcriptome sequencing. Total RNA was extracted using the standard method, and the integrity and total amount of RNA were detected by Agilent 2100 bioanalyzer (Agilent Technologies, CA, USA). Then, the library construction and quality inspection, sequencing, data quality control, gene expression level quantification and differential expression analysis were carried out in turn. The detailed method follows those described by Yang, et al. [34].

### Statistical analysis

Statistical analysis was performed using IBM SPSS Statistics software. The experimental data were analyzed by analysis of variance or student’s t-test.

## Data Availability

No datasets were generated or analysed during the current study.

## References

[1] Chohan S, Perveen R, Abid M, Tahir MN, Sajid M. Cotton diseases and their management. Cotton production and uses: agronomy, crop protection, and postharvest technologies, 2020; 239–270.

[2] Yadav R, Bunker RN, Sharma SS, Trivedi A, Rawal P. Survey, incidence and integrated disease management of cotton root rot caused by *Rhizoctonia solani* (Kuhn). J Pharm Innov. 2022;11(8):1618–21.

[3] Pacios-Michelena S, Aguilar Gonzalez CN, Alvarez-Perez OB, Rodriguez-Herrera R, Chávez-González M, Arredondo R, Ilyina A. Application of *Streptomyces* antimicrobial compounds for the control of phytopathogens. Front Sustain Food S. 2021;5:696518.

[4] Boruta TA. Bioprocess perspective on the production of secondary metabolites by *Streptomyces* in submerged co-cultures. World J Microb B. 2021;37(10):171. 10.1007/s11274-021-03141-z PMC8421279 34490503

[5] Chouyia FE, Ventorino V, Pepe O. Diversity, mechanisms and beneficial features of phosphate-solubilizing *Streptomyces* in sustainable agriculture: a review. Front Plant Sci. 2022;13:1035358. 36561447 10.3389/fpls.2022.1035358 PMC9763937

[6] Wang M, Li H, Li J, Zhang W, Zhang J. *Streptomyces* Straintheir metabolitesolites for Biocontrol of Phytopathogens in Agriculture. J Agric Food Chem. 2024;72(4):2077–88. 38230633 10.1021/acs.jafc.3c08265

[7] Olanrewaju OS, Babalola OO. *Streptomyces*: implications and interactions in plant growth promotion. Appl Microbiol Biotechnol. 2019;103(3):1179–88. 30594952 10.1007/s00253-018-09577-y PMC6394478

[8] Plackett RL, Burman JP. The design of optimum multifactorial experiments. Biometrika. 1946;33(4):305–25.

[9] Ramírez-López C, Chairez I, Fernández-Linares L. A novel culture medium designed for the simultaneous enhancement of biomass and lipid production by *Chlorella vulgaris* UTEX 26. Bioresour Technol. 2016;212:207–16. 27099946 10.1016/j.biortech.2016.04.051

[10] Kong Y, Zou P, Miao L, Qi J, Song L, Zhu L, Xu X. Medium optimization for the production of anti-cyanobacterial substances by *Streptomyces sp.* HJC-D1 using response surface methodology. Environ Sci Pollut Res Int. 2014;21(9):5983–90. 24464079 10.1007/s11356-014-2532-5

[11] Noman E, Al-Gheethi AA, Talip BA, Mohamed R, Kassim AH. Oxidative enzymes from newly local strain *aspergillus iizukae* EAN605 using pumpkin peels as a production substrate: optimized production, characterization, application and techno-economic analysis. J Hazard Mater. 2020;386:121954. 31884363 10.1016/j.jhazmat.2019.121954

[12] Park YS, Kang SW, Lee JS, Hong SI, Kim SW. Xylanase production in solid state fermentation by *Aspergillus Niger* mutant using statistical experimental designs. Appl Microbiol Biotechnol. 2002;58(6):761–6. 12021796 10.1007/s00253-002-0965-0

[13] Nguyen HP, T, Morançais M, Fleurence J, Dumay J. *MastocStellatusllatus* as a source of R-phycoerythrin: optimization of enzyme assisted extraction using response surface methodology. J Appl Phycol. 2017;29:1563–70.

[14] Shao SN, Zhang AQ, Baerna K, Zhang GQ. The control effect of *actinomycete* strain KN37 against Tomato Gray Mold. J Trop Biology. 2019;10:258–63.

[15] Zhao J, Li Q, Zeeshan M, Zhang GQ, Wang CJ, Han XQ, Yang DS. Integrative Genomics and Bioactivity-Guided Isolation of Novel Antimicrobial Compounds from *Streptomyces sp.* KN37 in Agricultural Applications. Molecules. 2024;29(9):2040. 38731531 10.3390/molecules29092040 PMC11085741

[16] Dandare SU, Håkansson M, Svensson LA, Timson DJ, Allen CCR. Expression, purification and crystallization of a novel metagenome-derived salicylaldehyde dehydrogenase from Alpine soil. Acta Crystallogr F Struct Biol Commun. 2022;78(Pt 4):161–9. 35400668 10.1107/S2053230X22002345 PMC8996149

[17] Ruiz-Villafán B, Cruz-Bautista R, Manzo-Ruiz M, Passari AK, Villarreal-Gómez K, Rodríguez-Sanoja R, Sánchez S. Carbon Catabolite regulation of secondary metabolite formation, an old but not well-established regulatory system. Microb Biotechnol. 2022;15(4):1058–72. 33675560 10.1111/1751-7915.13791 PMC8966007

[18] Ibrahim HM, Elkhidir EE. Response surface method as an efficient tool for medium optimisation. Trends Appl Sci Res. 2011;6(2):121.

[19] Zhou Y, Sun YB, He HW, Feng JT, Zhang X, Han LR. Optimization of medium compositions to improve a novel glycoprotein production by *Streptomyces kanasenisi* ZX01. AMB Express. 2017;7(1):6. 28050846 10.1186/s13568-016-0316-7 PMC5209317

[20] Liu E, Li M, Abdella A, Wilkins MR. Development of a cost-effective medium for submerged production of fungal aryl alcohol oxidase using a genetically modified *aspergillus nidulans* strain. Bioresour Technol. 2020;305:123038. 32120232 10.1016/j.biortech.2020.123038

[21] Liu H, Zhang D, Zhang X, Zhang X, Zhou C, Zhou P, Zhi Y. Medium optimization for spore production of a straw-cellulose degrading actinomyces strain under solid-state fermentation using response surface method. Sustainability. 2020;12(21):8893.

[22] Usha Kiranmayi M, Sudhakar P, Sreenivasulu K, Vijayalakshmi M. Optimization of Culturing Conditions for Improved Production of Bioactive Metabolites by *Pseudonocardia sp.* VUK-10. Mycobiology. 2011;39(3):174–81. 22783100 10.5941/MYCO.2011.39.3.174 PMC3385111

[23] Wang H, Zhou Y, Xu S, Zhang B, Cernava T, Ma Z, Chen Y. Enhancement of herbicolin A production by integrated fermentation optimization and strain engineering in *Pantoea agglomerans* ZJU23. Microb Cell Fact. 2023;22(1):50. 36915090 10.1186/s12934-023-02051-z PMC10012537

[24] Tanner RS. Cultivation of bacteria and fungi. Man Environ Microbiol, 2007: 69–78.

[25] Kontro M, Lignell U, Hirvonen MR, Nevalainen A. pH effects on 10 *Streptomyces sp.* growth and sporulation depend on nutrients. Lett Appl Microbiol. 2005;41(1):32–8. 15960749 10.1111/j.1472-765X.2005.01727.x

[26] Yun TY, Feng RJ, Zhou DB, Pan YY, Chen YF, Wang F, Yin LY, Zhang YD, Xie JH. Optimization of fermentation conditions through response surface methodology for enhanced antibacterial metabolite production by *Streptomyces sp.* 1–14 from cassava rhizosphere. PLoS ONE. 2018;13(11):e0206497. 30427885 10.1371/journal.pone.0206497 PMC6241123

[27] Patel G, Khobragade TP, Avaghade SR, Patil MD, Nile SH, Kai G, Banerjee UC. Optimization of media and culture conditions for the production of tacrolimus by *Streptomyces tsukubaensis* in shake flask and fermenter level. Biocatal Agri Biotech. 2020;29:101803.

[28] Talukdar S, Talukdar M, Buragohain M, Yadav A, Yadav RN, Bora TC. Enhanced candicidal compound production by a new soil isolate *Penicillium Verruculosum* MKH7 under submerged fermentation. BMC Microbiol. 2016;16(1):288. 27938325 10.1186/s12866-016-0713-8 PMC5225592

[29] Duan Y, Chen J, He W, Chen J, Pang Z, Hu H, Xie J. Fermentation optimization and disease suppression ability of a *Streptomyces ma.* FS-4 from banana rhizosphere soil. BMC Microbiol. 2020;20(1):24. 32005152 10.1186/s12866-019-1688-z PMC6995205

[30] Korayem AS, Abdelhafez AA, Zaki MM, Saleh EA. Optimization of biosurfactant production by *Streptomyces* isolated from Egyptian arid soil using plackett–burman design. Ann Agri Sci. 2015;60(2):209–17.

[31] Bhaturiwala R, Bagban M, Mansuri A, Modi H. Successive approach of medium optimization using one-factor-at-a-time and response surface methodology for improved β-mannanase production from *Streptomyces Sp*. Bioresource Technol Rep. 2022;18:101087.

[32] Mu LY. Research method of plant chemical protection. Chin Agricultural Press, 1994; 76–82.

[33] Wang X, Zhi Y. Altered urinary metabolomics in Hereditary Angioedema. Metabolites. 2022;12(11):1140. 36422280 10.3390/metabo12111140 PMC9696332

[34] Yang K, Yang L, Fan W, Long GQ, Xie SQ, Meng ZG, Zhang GH, Yang SC, Chen JW. Illumina-based transcriptomic analysis on recalcitrant seeds of *Panax notoginseng* for the dormancy release during the after‐ripening process. Physiol Plant. 2019;167(4):597–612. 30548605 10.1111/ppl.12904

